# Editorial for the Molecular Mechanisms in Neurodevelopmental Disorders Special Issue

**DOI:** 10.3390/genes14091762

**Published:** 2023-09-04

**Authors:** Irene Madrigal

**Affiliations:** Hospital Clinic of Barcelona, Biochemistry and Molecular Genetics and Fundació de Recerca Clínic Barcelona—Institut d’Investigacions Biomèdiques August Pi i Sunyer (FRCB-IDIBAPS), 08036 Barcelona, Spain; imadbajo@clinic.cat

Neurodevelopmental disorders are a group of neurological disorders that may give rise to delayed or impaired cognition, communication, adaptive behavior, and psychomotor skills. Some neurodevelopmental disorders are intellectual disability, autism spectrum disorder, attention hyperactivity disorder, others. The etiology of these disorders is multifactorial and results from the reciprocal interaction of genetic and environmental factors. The genetic architecture is complex and heterogeneous and includes a wide range of alterations, such us copy number variants, single nucleotide variant, structural variants, epigenetic variants or immunological factors during pregnancy, among others. Despite multiple studies and research projects carried out worldwide, genetic diagnosis in patients with rare diseases takes many years and sometimes, after multiple tests, a definitive diagnosis is not reached. Successful implementation of genomic medicine hinges on accurate, evidence-based interpretation of genetic data to ensure both appropriate clinical management and care.

In this Special Issue, five research groups report new insights into the etiology of neurodevelopmental disorders. Two publications describe the results of the implementation of high-throughput technologies for the diagnosis of neurodevelopmental disorders. Massively parallel sequencing has become the standard technique for clinical practice. Specifically, whole exome sequencing (WES) has led to enormous progress in deciphering monogenic forms of various genetic diseases. Spataro et al. applied a gene panel testing including 460 dominant and X-linked genes to 398 patients affected by intellectual disability, global developmental delay and/or autism [1]. A molecular diagnosis was established in 28.6% of these patients. The authors conclude that, despite the high throughput of the panel, WES approaches may represent a more robust solution. In the other paper, Alvarez-Mora et al. applied WES as a first-line test in 209 patients with neurodevelopmental disorders and establish diagnostic rate around 32% [2]. The authors conclude that the implementation of WES for routine diagnosis improves the diagnostic process, making it faster and more efficient.

Nowadays, the functional consequences of many genetic variants associated to disease still remain unclear. Di Stazio et al., investigated the effect of two de novo *CSNK2B* variants in patients mainly presenting mild impairment of psychomotor development; low anthropometric values and epilepsy [3]. CSNK2B is responsible for Poirer-Bienvenu Neurodevelopmental Syndrome (POBINDS), and it encodes for the regulatory subunit of the casein kinase II, a serine/threonine kinase that is highly expressed in the brain. In this work they combined predictive functional and structural analysis and in vitro experiments such as plasmid constructs, co-immunoprecipitation or Actinomycin D treatment, among others, in order to investigate the effect of these CSNK2B variants. Results indicated that the loss of the CK2beta protein, due to the instability of mutant CSNK2B mRNA and protein, resulted in a reduced amount of CK2 complex and affected its kinase activity, and may be underlie the POBINDS phenotype. Other functional studies have been developed by the group of Birkhoff with the transcription factor Zeb2 (Birkhoff et al., 2023) [4]. Zeb2 is a transcription factor bind to DNA to two separated E-box like sequences and functions as a DNA-binding transcriptional repressor that interacts with activated SMADs and interacts with the nucleosome remodeling and histone deacetylation complex (Verstappen et al., 2008) [5]. The transcription factor Zeb2 controls cell fate decisions, differentiation, and/or maturation in multiple cell lineages in embryos and after birth. Mutations in ZEB2 cause Mowat-Wilson syndrome (Mowat et al., 2003; Ishihara et al., 2004) [6,7], a rare congenital disease. By using chromatin immunoprecipitation coupled with sequencing, Birkhoff et al., detected a new, major binding site that maps promoter-proximal to Zeb2 itself. The homozygous deletion of this site demonstrates that autoregulation of Zeb2 is necessary to elicit the appropriate Zeb2-dependent effects in embryonic stem cells-to-neuroprogenitor cells differentiation. In addition, this study also contributes to explain developmental disorders caused by ZEB2 deficiency, including Mowat-Wilson syndrome.

Finally, another factor that has been related to the development of neurodevelopmental syndromes is placental immune responses. The placental immune response affects the outcome of the pregnant woman’s susceptibility to certain infectious diseases. Viral or bacterial infections during pregnancy have been associated with important qualitative immunological changes (i.e., Abu-Raya et al., 2020) [8]. In particular, the possible contribution of prenatal programming through placental effects as an underlying mechanism that links to neurodevelopmental disorders risk, susceptible to environmental stressors (Hall et al., 2023; Woods et al., 2023) [9,10]. In fact, maternal immune activation is associated with changes in gene expression in the brain [11]. Understanding of alterations in myelin sheath formation and neuron-to-neuron signal transmission in behavioral disorders associated with maternal immune activation could be improved by detecting changes in the molecular structure of the gland.

The articles included in this Special Issue provide comprehensive related to neurodevelopmental disorders and we hope this issue will help researchers search for additional genetic associations that will help refine our understanding of the etiology of neurodevelopmental disorders.

## Data Availability

Not applicable.
